# Vector competence of *Aedes aegypti* and screening for differentially expressed microRNAs exposed to Zika virus

**DOI:** 10.1186/s13071-021-05007-7

**Published:** 2021-09-27

**Authors:** Chunling Zhu, Yuting Jiang, Qianghui Zhang, Jian Gao, Chaojie Li, Chunxiao Li, Yande Dong, Dan Xing, Hengduan Zhang, Teng Zhao, Xiaoxia Guo, Tongyan Zhao

**Affiliations:** 1 Department of Vector Biology and Control, State Key Laboratory of Pathogen and Biosecurity, Beijing Key Laboratory of Vector Borne and Natural Focus Infectious Diseases, Beijing Institute of Microbiology and Epidemiology, Beijing, 100071 China; 2 Department of Clinical Laboratory, Guangxi International Zhuang Medicine Hospital, Nanning, 530201 Guangxi China

**Keywords:** Zika virus, *Aedes aegypti*, Vector competence, microRNA

## Abstract

**Background:**

Zika virus (ZIKV) is transmitted to humans primarily by *Aedes aegypti*. Previous studies on *Ae. aegypti* from Jiegao (JG) and Mengding (MD) in Yunnan province, China have shown that these mosquitoes are able to transmit ZIKV to their offspring through vertical transmission, indicating that these two *Ae. aegypti* strains pose a potential risk for ZIKV transmission. However, the vector competence of these two *Ae. aegypti* strains to ZIKV has not been evaluated and the molecular mechanisms influencing vector competence are still unclear.

**Methods:**

*Aedes aegypti* mosquitoes from JG and MD were orally infected with ZIKV, and the infection rate (IR), dissemination rate (DR), transmission rate (TR) and transmission efficiency (TE) of these two mosquito strains were explored to evaluate their vector competence to ZIKV. On 2, 4 and 6 days post-infection (dpi), the small RNA profiles between ZIKV-infected and non-infected *Ae. aegypti* midgut and salivary gland tissues were compared to gain insights into the molecular interactions between ZIKV and *Ae. aegypti*.

**Results:**

There were no significant differences in the IR, DR, TR and TE between the two *Ae. aegypti* strains (*P* > 0.05). However, ZIKV RNA appeared 2 days earlier in saliva of the JG strain, which indicated a higher competence of the JG strain to transmit ZIKV. Significant differences in the microRNA (miRNA) expression profiles between ZIKV-infected and non-infected *Ae. aegypti* were found in the 2-dpi libraries of both the midgut and salivary gland tissues from the two strains. In addition, 27 and 74 miRNAs (|log2 fold change| > 2) were selected from the miRNA expression profiles of ZIKV-infected and non-infected midgut and salivary gland tissues from the JG and MD strains, respectively.

**Conclusions:**

Our results provide novel insights into the ZIKV–mosquito interactions and build a foundation for future research on how miRNAs regulate the vector competence of mosquitoes to this arbovirus.

**Graphical abstract:**

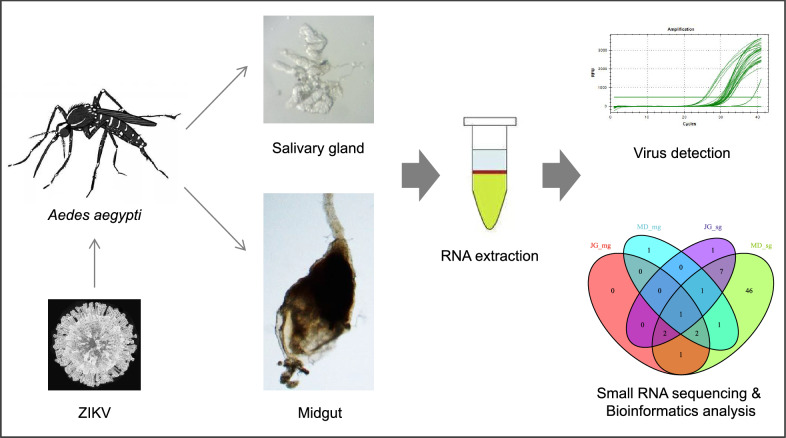

**Supplementary Information:**

The online version contains supplementary material available at 10.1186/s13071-021-05007-7.

## Background

Zika virus (ZIKV) (family Flaviviridae, genus* Flavivirus*) is a single-stranded RNA virus that is primarily transmitted by *Aedes aegypti*. ZIKV was discovered initially from a sentinel monkey in Uganda, Africa, in 1947 [1, 2] and has since diverged into two main genotypes: African and Asian genotypes [3]. The first case of human infection in Brazil was recorded in 2015 [4, 5]. ZIKV continues to spread, currently recorded in 86 countries or regions around the world, and it is estimated that approximately 3.6 billion people currently live in areas at risk of transmission [6]. ZIKV can cause Guillain-Barré syndrome (an acute inflammatory immune-mediated polyneuropathy) in adults and fetal microcephaly in newborns [7]. The spread of ZIKV in the Western Hemisphere poses a major threat to public health. Currently, there are no specific drugs or vaccines against ZIKV infection, with vector control being the only viable method for alleviating the diseases caused by this virus. Genetic manipulation of vectors to modulate vector competence is also a potential novel approach for controlling vector-borne diseases [8–10]. Therefore, knowledge of molecular interactions between viruses and mosquito hosts is critical to manipulating vector competence.

Vector competence to arboviruses is affected by both intrinsic factors and the molecular mechanism in mosquitoes. When a female mosquito ingests an infectious blood meal, the first organ that an arbovirus comes into contact with is the midgut of the mosquito. The arbovirus then enters the hemolymph and spreads to secondary tissues, such as the fat body, trachea and salivary gland. Finally, the virus is released into salivary tubes and transmitted to uninfected vertebrate hosts by horizontal transmission [11]. Therefore, in order to infect a vector, the virus must overcome the tissue barriers associated with the midgut and salivary gland and cope with the related antiviral pathways. In this context, exploration of the interactions of virus and tissue barriers to arbovirus infection may provide interesting results.

Mosquitoes possess a few antiviral pathways that are able to limit the spread of virus, such as reactive oxygen species (ROS) production and the innate immune pathway. However, these approaches appear to be virus specific. It has been shown that the antioxidants in the midgut induced by catalase are triggered after the mosquito has ingested a blood meal and that this state of antioxidation can increase mosquito susceptibility to dengue virus (DENV), but does not affect ZIKV infection [12]. In the mosquito’s innate immune pathways, the Toll and JAK-STAT pathways are related to the regulation of DENV infection, yet they do not appear to influence other arboviruses, such as chikungunya virus (CHIKV) or ZIKV [13–16]. The RNA interference (RNAi) pathway is also an important way to determine the interactions between arboviruses and their mosquito hosts. The RNAi pathway mainly includes three main small RNA pathways: small interfering RNA (siRNA), microRNA (miRNA) and P-element-induced wimpy testis-interacting RNA (piRNA) [17]. Although miRNAs could potentially be involved in ZIKV–mosquito interactions, the direct interaction and specific mechanism are still unclear [18].

miRNAs are endogenous small non-coding RNAs of 21–24 nucleotides (nt) that regulate gene expression through binding to the target mRNA and initiating mRNA degradation by the RNA-induced silencing complex (RISC) [19]. In mosquitoes, miRNAs are involved in the replication of virus by regulating host factors. Some studies have indicated that DENV [20] and ZIKV [18] infections could alter miRNA profiles in mosquitoes. The possibility that some miRNAs in *Aedes* may affect viral replication has also been reported. In one study, the miRNA miR-252 in *Ae. albopictus* increased after a DENV-infected blood meal, and inhibition of the miRNA enhanced viral replication while overexpression of the miRNA limited viral replication [21]. Another study demonstrated that aae-miR-375 could enhance DENV serotype 2 infection in an *Ae. aegypti* cell line [22]. These studies indicate that miRNAs play an important regulatory role in the interactions between arboviruses and mosquitoes, which may affect the vector competence of mosquitoes. It is possible that more obvious changes in miRNAs could be observed in specific tissues infected with virus.

*Aedes aegypti* is an important invasive species in Yunnan province in China since 2002 [23]. Yunnan province is the main passageway for China to connect to Southeast Asia, and Jiegao and Mengding are two important trade ports in this province. To date, there has been no study on the risk of a ZIKV epidemic through mosquitoes in these two cities. In the present study, we collected two *Ae. aegypti* strains in Jiegao and Mengding to assess their vector competence to ZIKV. We also analyzed the results of high-throughput sequencing of microRNAs in the midgut and salivary gland infected with ZIKV to explore the molecular interactions between the mosquito host and the virus, which could contribute to development of new insect-borne disease prevention strategies.

## Methods

### Virus strain

The viral strain used in this study was ZIKV SZ01 strain obtained from the Microbial Culture Collection Center of the Beijing Institute of Microbiology and Epidemiology. This virus was originally isolated from a patient who returned from Samoa to China in 2016 (GenBank Nos. KU866423) [24]. The virus has been maintained in the *Aedes albopictus* C6/36 cell lines and was passaged four times before the study.

### Infection of mosquitoes

Mosquito larvae and pupae of two *Ae. aegypti* mosquito strains were originally collected from Jiegao (23°58′40″N, 97°53′ 24″E) and Mengding (23°33′00″N, 99°3′33″E), Yunnan Province, China, respectively, in July 2018. All larvae and pupae were reared to adult forms in the laboratory under the same conditions (26 ± 1 °C, relative humidity [RH] 75 ± 5%, a 14:10-h light/dark [L/D] photoperiod). Adult mosquitoes were provided with 10% sugar water. The fourth generation of two *Ae. aegypti* stains were used to perform ZIKV oral infection experiments. Detailed methods of mosquito collection and husbandry are described in our previous work [25].

ZIKV suspension mixed with mouse blood (1:1) were provided to 5-day-old female mosquitoes for oral feeding using the Hemotek membrane feeding system (Sihuan, Beijing, China) to keep the virus blood meal at 37 °C. The virus titer of the virus blood meal was determined to be 1.5 × 10^4^ plaque-forming units (PFU)/ml at the time of feeding, using the plaque assay with the BHK-21 cell line (ATCC Number: CCL-10) . After 1 h of blood-feeding, fully engorged females were transferred to plastic cups (10–15 mosquitoes/tube) and reared at 29 ± 1 °C and 75 ± 5% RH under a 14/10-h L/D cycle. A 5% sucrose solution on cotton pads was provided to these mosquitoes.

### Vector competence indices

For each mosquito examined, the midgut, salivary gland and saliva were tested separately to analyze vector competence in the two *Ae. aegypti* strains at 2, 4, 6, 8, 10, 14 and 20 days post-infection (dpi), and 10 mosquitoes were used per time point. The experiments were performed three times independently, except for those carried out on 20 dpi that were performed two times. The midgut and salivary gland of each mosquito were dissected and washed 3 times in phosphate-buffered saline and transferred to 1.5-ml microtubes containing 100 μl of Dulbecco’s modified Eagle’s medium (DMEM; GIBCO™, Invitrogen, Beijing, China) supplemented with 2% fetal bovine serum (FBS). Saliva was collected from individual mosquitoes at 2, 4, 6, 8, 10, 14 and 20 dpi using the method described by Dubrulle et al. [26]. After removing the wings and legs of each mosquito, the proboscis was inserted into a glass capillary containing 5 μl of FBS. After 30 min, the FBS containing saliva was mixed with 100 μl of DMEM in 1.5-ml microtubes.

The ZIKA RNA of these samples was extracted using the QIAamp Viral RNA Mini kit (Qiagen, Hilden, Germany) and detected using a commercial kit (Zika virus nucleic acid detection kit; Daan Gene Co. Ltd., Guangdong, China; Cat no: DA0711). The procedures are described in detail in our previous study [25].

Vector competence of the mosquitoes was evaluated by calculating the infection rate (IR; number of positive midgut/the total number of mosquitoes tested), dissemination rate (DR; number of infected salivary gland/the number of infected midgut), transmission rate (TR; number of infected saliva/the number of infected salivary gland) and transmission efficiency (TE; number of infected saliva/the total number of mosquitoes tested).

All statistical analyses were conducted using SPSS version 21.0 software (SPSS IBM Corp., Armonk, NY, USA). The IR, DR, TR and TE of two *Ae. aegypti* strains were compared using Fisher’s exact test. Viral titers were compared with the Mann–Whitney U-test to determine differences between two strains at different times. *P*-values < 0.05 were considered to be significant.

### Viability of ZIKV in saliva of *Ae. aegypti*

Saliva samples were collected from JG and MD *Ae. aegypti* strains (30 individuals of each strain) on 8 dpi using the method described by Dubrulle et al. [26]. In brief, wings and legs of each mosquito were removed and the proboscis was inserted into a glass capillary containing 5 μl of FBS. After 30 min of salivation, FBS containing saliva collected from 30 individual mosquitoes was mixed with 200 μl of RPMI Medium 1640 (GIBCO™, Invitrogen, Beijing, China). The mixture was then inoculated into one well of a 12-well plate covered with a single layer of C6/36 cells for 2 h. After the incubation period, the mixture was removed and 1 ml RPMI Medium 1640 (containing 2% FBS, 1% 100U/ml penicillin and 100 ug/ml streptomycin) was added into the well. The plate was maintained in a cell incubator at 28 °C and 5% CO_2_ for 7 days. Cells were observed by inverted microscopy every day to examine the cytopathic effect (CPE).

### Sample preparation and RNA extraction

Five-day-old female mosquitoes from the two strains (JG and MD) were fed with ZIKV-infected blood meal (1.5 × 10^4^ PFU/ml) or a blood meal devoid of ZIKV. The infection procedure was the same as described for the vector competence test. The midgut and salivary gland of approximately 100 mosquitoes from each of the groups (infected group and control group) of the two strains were collected by dissecting individual mosquitoes on 2, 4 and 6 dpi. These samples were collected separately in 1.5-ml RNase-free microcentrifuge tubes containing 500 μl TRIzol reagent (Invitrogen, Thermo Fisher Scientific, Waltham, MA USA) and stored at − 80 °C until subsequent RNA extraction. Total RNA was extracted from 24 groups using the TRIzol reagent according to the manufacturer’s protocol. The quality and quantity of RNA were measured by the Agilent 2100 Bioanalyzer System (Agilent Technologies, Inc., Santa Clara, CA, USA). Each RNA sample was divided to two parts, with one used for small RNA library preparation and sequencing and the second part used for reverse transcription-quantitative PCR (RT-qPCR) analysis.

### Small RNA library preparation and sequencing

The RNA extraction, library preparation and sequencing analyses were performed by the BGI Company (Shenzhen, China). Briefly, 1 µg of each pooled total RNA was used to create small RNA libraries. RNA fragments (18–30 nt) were isolated from total RNA by polyacrylamide gel electrophoresis (PAGE). Small RNA libraries were prepared in accordance with the manufacturer’s instructions (Illumina, San Diego, CA, USA). Library creation uses a sequential addition of first a 3′ adapter sequence followed by a 5′ adapter sequence. A complimentary DNA (cDNA) copy was then synthesized using ProtoScript reverse transcriptase (New England Biolabs, Ipswich, MA, USA) and a primer complimentary to a segment of the 3′ adapter. The cDNA was amplified using reverse transcriptase PCR (RT-PCR) for 12–15 PCR cycles to complete the libraries. The quality of cDNA was checked using the Agilent 2100 Bioanalyzer system (Agilent Technologies Inc.). Libraries were then submitted to BGI (Shenzhen, China) for small RNA sequencing (RNA-seq) using the Illumina genomic analyzer.

### Bioinformatics

The Fastx toolkit was used to remove adapter sequences and reads with low-quality scores from datasets; Bowtie2 was used to map clean reads to the reference genome and to other sRNA databases; and cmsearch was used for searching the Rfam database. After removing the adapter and low-quality sequences, all clean small RNA tags were matched with the NCBI GenBank (http://www.ncbi.nlm.gov/) ribosome RNA (rRNA), small cytoplasmic RNA (scRNA, small nucleolar RNA (snoRNA), small nuclear RNA (snRNA) and transfer RNA (tRNA) databases, and the matched tags were removed from the unlabeled tags. In order to ensure that every small RNA mapped to only one annotation, small RNA was classified and annotated according to the priority rule of all rRNA (in which GenBank > Rfam > repeat > exon > intron > known miRNA) [27]. Mature and pre-miRNA sequences of *Ae. aegypti* (AaegL5.0) were used as a reference miRNA from the miRBase v.21 database. The unmatched clean reads were used for the prediction of novel miRNAs by MiRDeep2 by exploring their secondary structure, information of Dicer cleavage sites and energy. RNA secondary structures were predicted by RNA-fold (http://rna.tbi.univie.ac.at/).

### Expression profiling of miRNAs in response to ZIKV

An algorithm was used to identify differentially expressed miRNAs between ZIKV-infected samples and control samples. *p*(χ) = e^−λ^λ^χ^ / χ!, where χ is defined as the number of reads from small RNA and λ is the real transcripts of the miRNA. The method is described in detail by Audic et al. [28]. When the false discovery rate (FDR) was < 0.001, changes in known miRNA expression between infected samples and control samples were considered to be significant. miRNAs with log2 fold change (FC) > 1 were designated as being significantly upregulated, and miRNAs with log2 FC ≤1 were designated as being significantly downregulated.

### RT-qPCR analysis of miRNAs

The expression of miRNAs was validated by two-step RT-qPCR using the primers listed in Additional file 1: Table S1. The RT reaction was conducted in a mixture containing 2 μl of dNTP (2.5 mM each), 2 μl of 10× RT Buffer, 2 μl of RT primer (10 μM), 8 μl of total RNA, 1 μl of RT enzyme (10 U/ul), 1 μl of RNase inhibitor (40 u/ul) and 4 μl of RNase free water, at 16 °C for 30 min, 42 °C for 40 min and 85 °C for 5 min. All RT reagents were purchased from Invitrogen (Carlsbad, CA, USA). The resulting cDNAs were used as templates for the qPCR reaction, which contained 8 μl of 2× PCR mix (Lot: 204145; Qiagen), 0.2 μl of each forward and reverse primers (10 μM), 1 μl of template and 6.6 μl of nuclease water. Amplification reactions were performed in an ABI ViiA 7 Real-Time PCR System (Applied Biosystems, Thermo Fisher Scientific, Waltham, MA, USA) programed as follows: 1 cycle at 95 °C for 2 min; then 94 °C for 10 s and 40 cycles at 55 °C for 10 s and 72 °C for 40 s. The analysis of relative expression levels of miRNAs was performed using 2^−∆∆CT^ method [29]. Small nuclear RNA (RNU6B) was used to normalize the FC in miRNA expression in each sample.

### Target prediction of miRNAs and functional analysis

The target genes of the differentially expressed miRNA were predicted using miRanda (http://www.microrna.org/microrna/home.do) and RNAhybrid (http://bibiserv.techfak.uni-bielefeld.de/rnahybrid/). The prediction of the functions of these miRNAs or their targets based on Gene Ontology (GO; http://amigo.geneontology.org/amigo) and the Kyoto Encyclopedia of Genes and Genomes (KEGG; https://www.genome.jp/kegg/pathway.html) pathway enrichment analysis.

## Results

### Vector competence to ZIKV in two *Ae. aegypti* strains

**Fig. 1:**
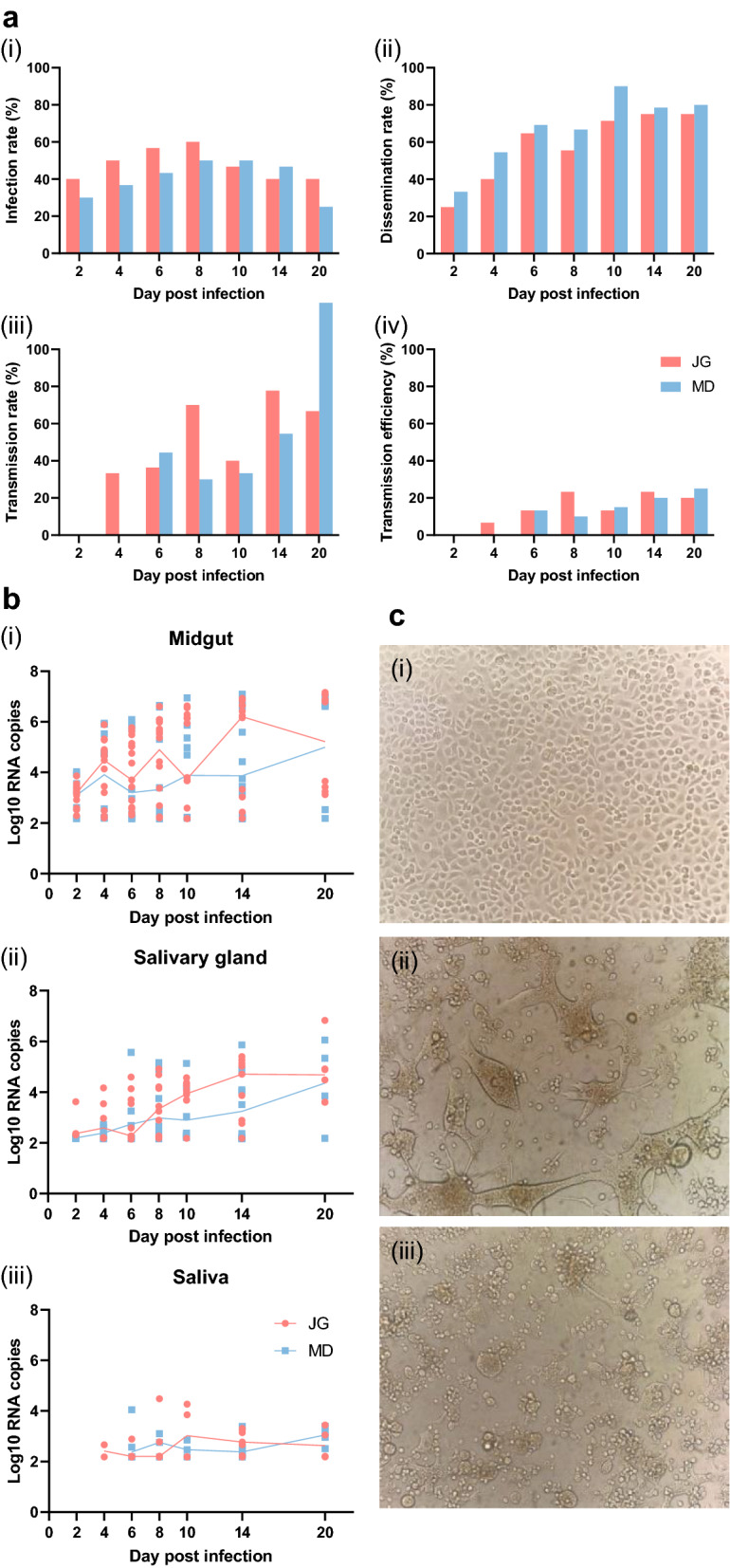
Vector competence to ZIKV in two *Aedes aegypti* strains. **a** Infection rate (IR), dissemination rate (DR), transmission rate (TR) and transmission efficiency (TE) of two *Ae. aegypti* strains to ZIKV. A total of 30 mosquitoes were analyzed per time point, except for 20 dpi (20 individuals). **b** Viral RNA copies in the midgut, salivary gland and saliva of the JG and MD *Ae. aegypti* strains at different time points after ZIKV infection. The line represents the median of results at each time point. **c** Normal C6/36 cells (**i**) or C6/36 cells inoculated for 7 days with saliva from JG (**ii**) or MD (**iii**) mosquito strains infected with ZIKV (×100 magnification). The results shown are combined results from three independent experiments. The IR, DR, TR and TE of the two *Ae. aegypti* strains were compared using Fisher’s exact test. Viral titers were compared using the Mann–Whitney U-test to determine differences between the two strains at different times. Abbreviations: JG, Jiegao strain; MD, Mengding strain

To evaluate the vector competence of *Ae. aegypti* (JG and MD strains) to ZIKV, we assessed the IR, DR, TR and TE. ZIKV RNA copies in the mosquito midgut, salivary gland and saliva were detected on 2, 4, 6, 8, 10, 14 and 20 dpi. ZIKV particles secreted in saliva were detected on 8 dpi. From 2 dpi onward, the midgut and salivary gland of each mosquito strain tested positive for ZIKV RNA. Overall, infection rates were moderate (40–60% for JG, 25–50% for MD). The DR were around 27–75% in the JG strain and 33–90% in the MD strain from 2 to 20 dpi, increasing with increasing time from initial infection. Between 14 and 20 dpi, the DR and TR remained high in the two *Ae. aegypti* strains (DR: 75–80%, TR: 55–125%). The transmission efficiencies of the two *Ae. aegypti* strains ranged from 7 to 25%. There were no significant differences in the IR, DR, TR and TE between the two *Ae. aegypti* strains at any time point during the experiments (Fisher’s exact test, *P* = 0.179 ~ 1.000; Fig. 1a). ZIKV RNA appeared 2 days earlier in saliva of the JG strain compared to the MD strain [4 vs 6 dpi; Fig. 1a (iv)], which indicates a higher vector competence of the JG strain to ZIKV.

The number of ZIKV RNA copies (log10 transformation) increased in the midgut and salivary gland of each mosquito strain over time, reaching 5.17 ± 0.89 and 4.72 ± 0.52 in these two tissues on 20 dpi, respectively. Virus level was low in the saliva and remained between 2.35 and 3.12 during 6–20 dpi. There was no significant difference in virus RNA level between two strains at any time point during the experiments (Mann–Whitney U-test,* U* = 12.50 ~ 22.00, *P* = 0.125 ~ 0.748; Fig. 1b).

On 8 dpi, saliva samples collected from the ZIKA- infected JG and MD *Ae. aegypti* strains (30 individuals of each strain) were inoculated separately into C6/36 cells, and uninfected C6/36 cells were used as the control (Fig. 1c). On day 7, the CPE could be observed in the saliva cells inoculated with infected saliva but not in the control group.

### Illumina sequencing of small RNAs

**Fig. 2:**
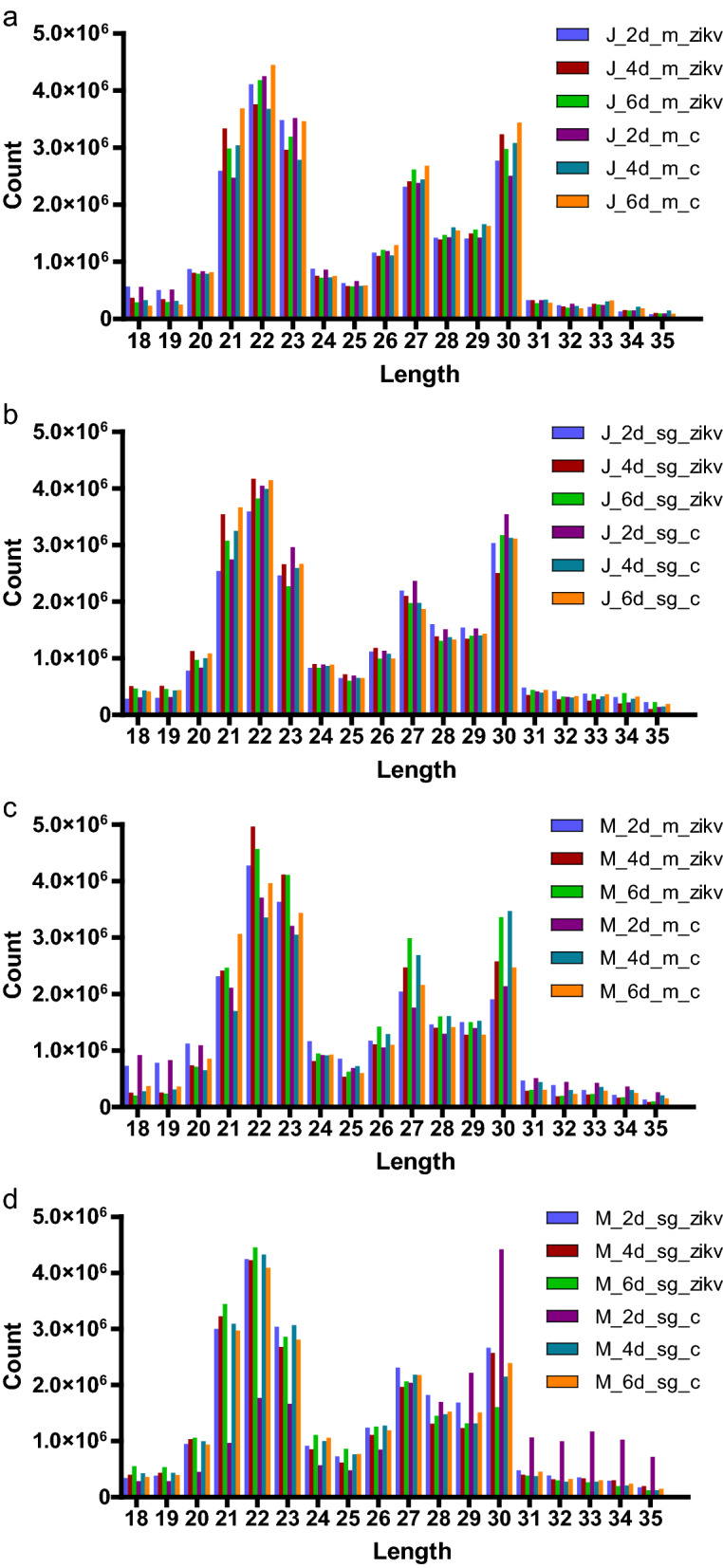
The length distribution of small RNAs from different samples. Mosquitoes of the two strains (J, Jiego strain; M, Mengding strain) fed either a ZIKA-infected blood meal (zikv group) or a non-infected blood meal [control group (c)] were dissected at the indicated time points [2, 4, 6 days after being fed the blood meal (2d, 4d, 6d, respectively)]. Different tissues (m, midgut; sg, salivary gland) were collected for RNA extraction and sequencing. Color-coded stacked bars are designated into four segments indicating the strain (J or M), the number of dpi (2d, 4d, 6d), tissue type (m or sg) and experimental group (zikv or c)

An Illumina sequencing platform was used to produce small RNA profiles of ZIKV-infected and non-infected *Ae. aegypti* mosquitoes (JG and MD strains, midgut and salivary gland). For the JG strain, we obtained 178 and 181 million combined raw reads from control and infected small RNA libraries, respectively; for the MD strain, 182 and 176 million reads were acquired from control and infected libraries, respectively (Additional file 2: Table S2). In order to obtain the clean reads, 9–33% of reads were discarded in different libraries due to their low-quality score or lack of adapter sequence. In those libraries, the small RNA length distribution showed a peak at 21–23 nt, representing the characteristic lengths of miRNAs and siRNAs. Another smaller peak was obtained at 27–30 nt, which may be related to piRNAs, a common feature of insect small RNA libraries (Fig. 2).

### Changes in *Ae. aegypti* miRNA expression profiles in response to ZIKV infection

**Fig. 3:**
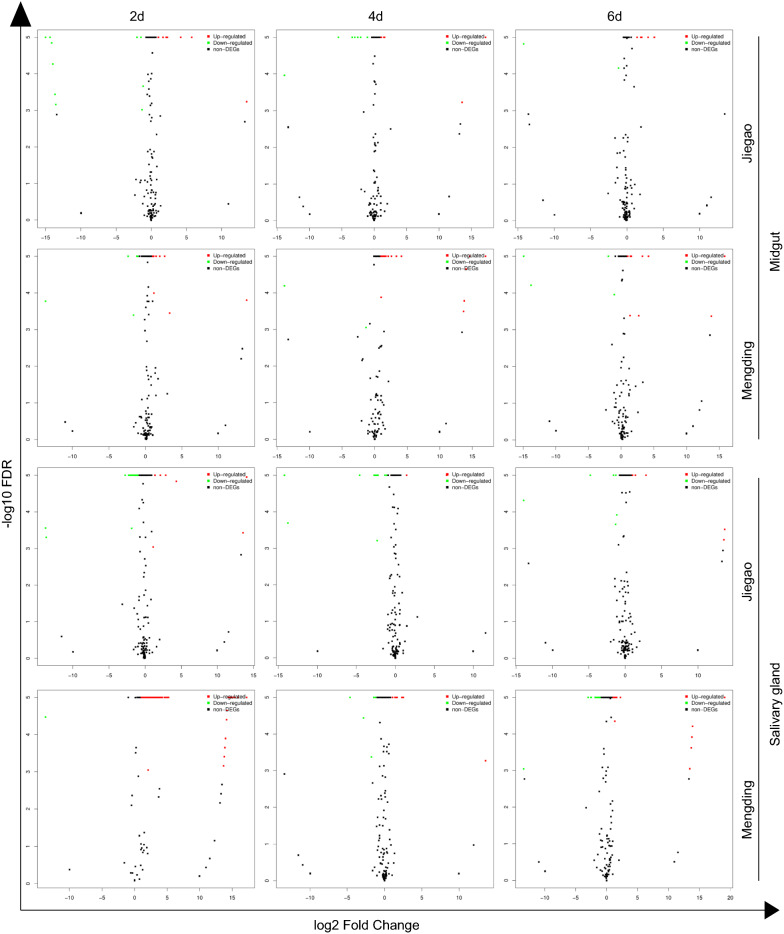
Volcano plots of miRNA expression levels. miRNA expression levels in the midgut or salivary gland of the two mosquito strains infected with ZIKV at the indicated time points are compared with those of control group. Those miRNAs with |log2 FC| > 1 and FDR < 0.001 (− log10 FDR > 3) were considered to be significantly changed. Red and green plots indicate significantly upregulated and downregulated expression, respectively. Black plots (non-DEGs) indicate no significant difference between infected and control libraries

Small RNA library analysis of ZIKV-infected *Ae. aegypti* midgut and salivary gland tissues identified 87 differentially regulated miRNAs for the JG strain and 216 differentially regulated miRNAs for the MD strain at the different time points compared with non-infected controls (|log2 FC|> 1) (Fig. 3). Interestingly, the most alterations in the miRNA profile were found in the 2-dpi libraries of both tissues from two strains. In addition, 27 and 74 miRNAs were identified from the JG and MD strains, respectively, with a standard of |log2 FC|> 2 in expression between the ZIKV-infected and non-infected group. (Additional file 3: Table S3). Of these miRNAs, eight were upregulated and six were downregulated with statistical significance in the midgut tissue, and three were upregulated and 10 were downregulated with statistical significance in the salivary gland tissue of JG strain. However, the changes in miRNA expression in the midgut and salivary gland tissues of MD strain were predominately upregulated.

**Fig. 4:**
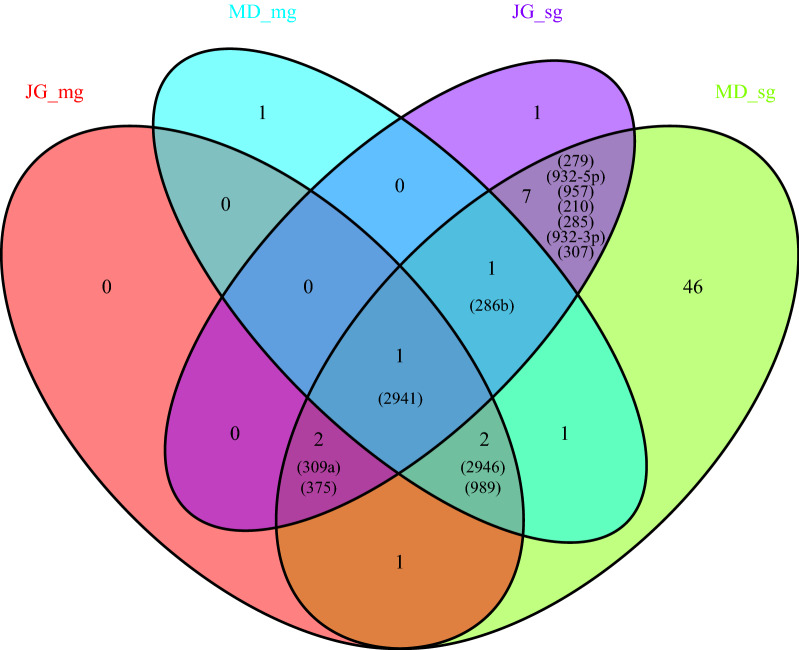
Venn diagram representing the number of differentially expressed miRNAs post-ZIKV infection between the two *Ae. aegypti* strains. The abbreviations of miRNAs that were expressed with |log2 FC| > 2 in both strains of *Ae. aegypti* mosquitoes are given in parentheses (e.g.: 2941 is the abbreviation of aae-miR-2941)

There were three miRNAs (aae-miR-2946, aae-miR-989, aae-miR-2941) in the midgut with |log2 FC| > 2 expressed in both the JG and MD strains of *Ae. aegypti* mosquitoes (Fig. 4; Table 1). These three miRNAs were upregulated in the midgut of the MD strain but downregulated in the midgut of the JG strain on 4 dpi and upregulated on 2 and 6 dpi. There were 11 miRNAs (aae-miR-2941, aae-miR-309a, aae-miR-375, aae-miR-286b, aae-miR-279, aae-miR-932-5p, aae-miR-957, aae-miR-210, aae-miR-285, aae-miR-932-3p, aae-miR-307) in the salivary gland with |log2 FC| > 2 expressed in both the JG and MD strains of *Ae. aegypti* mosquitoes (Fig. 4; Table 1). Among these 11 miRNAs, aae-miR-309a, aae-miR-286b and aae-miR-279 were upregulated in both strains; Aae-miR-307 was downregulated in both strains; and Aae-miR-932-5p, aae-miR-957, aae-miR-210, aae-miR-285 and aae-miR-932-3p were downregulated in the JG strain but upregulated in the MD strain.

**Table 1:**
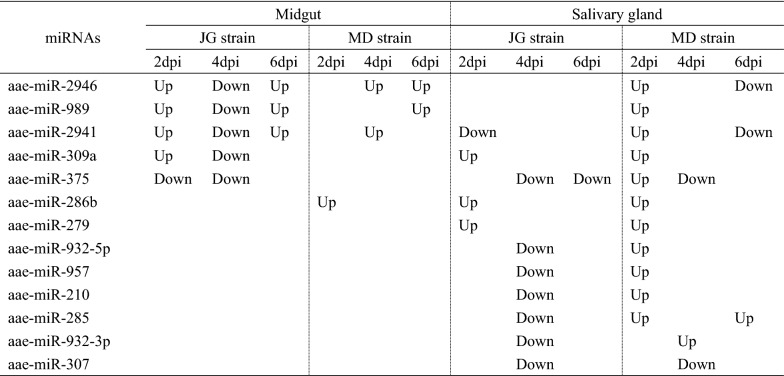
miRNA regulation in different tissues of *Ae. aegypti*

Down, Downregulated; Up, upregulated

### RT-qPCR validation of differentially expressed miRNAs

**Fig. 5:**
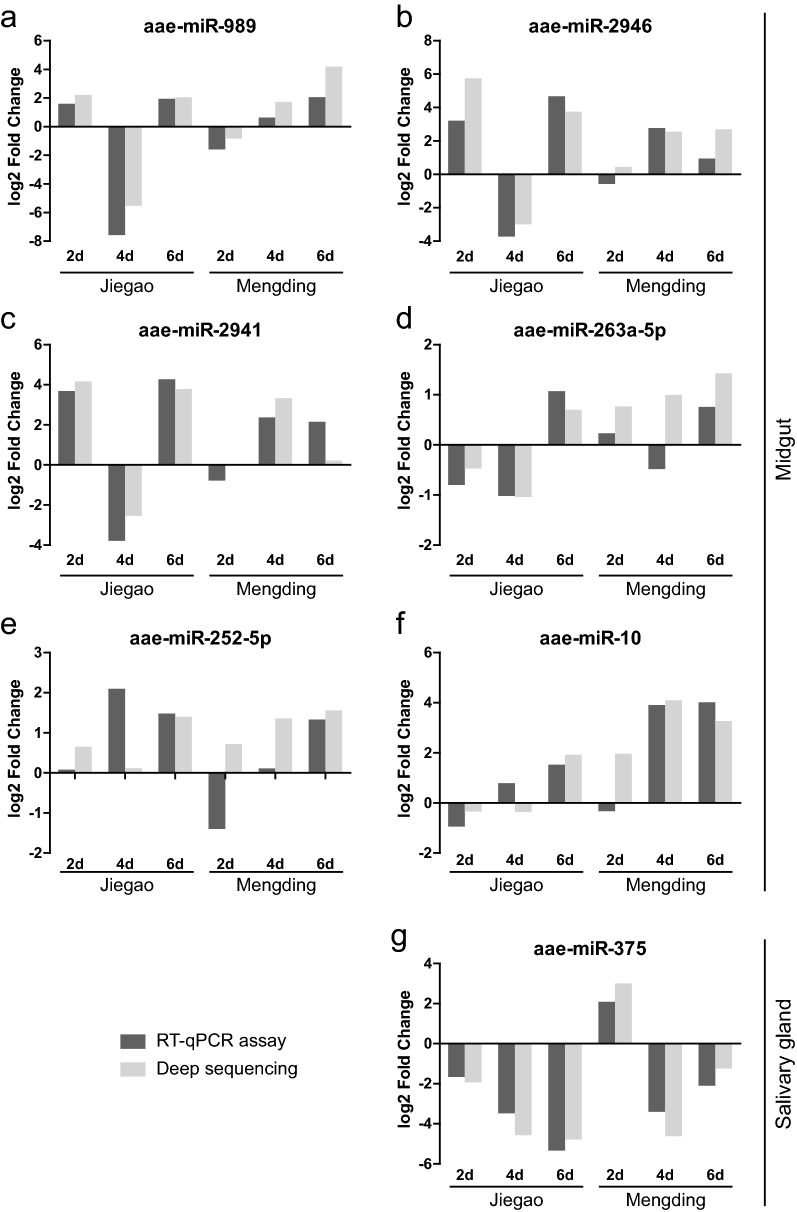
RT-qPCR verification of differentially expressed miRNAs from RNA-Seq. miRNA expression in the midgut (**a**–**f**) and salivary gland (**g**) of the JG or MD *Ae. aegypti* strains infected with ZIKV relative to the uninfected group at the indicated time point is shown. For the RT-qPCR assay, fold changes are averages of three technical replicates

To validate the results of deep sequencing, we determined the expression of seven miRNAs (aae-miR-989, aae-miR-2946, aae-miR-2941, aae-miR-263a-5p, aae-miR-252-5p, aae-miR-10, aae-miR-375), with RT-qPCR assays on 2, 4 and 6 dpi for both the JG and MD strains (Fig. 5). The results of these assays showed that aae-miR-2946 (2 dpi), aae-miR-2941 (2 dpi), aae-miR-263a-5p (4 dpi), aae-miR-252-5p (2 dpi), aae-miR-10 (2 dpi), all from the midgut of the MD strain, exhibited inconsistencies in expression, which were seen to be depleted by RT-qPCR but enriched by RNA-Seq. While aae-miR-10 (4 dpi) from the midgut of the JG strain was seen to be upregulated with the RT-qPCR assay, deep sequencing showed the opposite result. The expression levels of other miRNAs (36 out of 42 cases) obtained by RNA-Seq and RT-qPCR were consistent, indicating that the results from RNA-Seq were reliable.

### Target analysis of differentially abundant miRNAs

Elimination of duplicated miRNAs from the different groups resulted in 63 significantly differentially expressed miRNAs with |log2 FC|> 2. The targets of these miRNAs were predicted using the online software miRanda and RNAhybrid. These miRNAs had 4369 targets (Additional file 4: Table S4). The potential biological functions of these target genes were then explored usinge GO and KEGG functional enrichments.

**Fig. 6:**
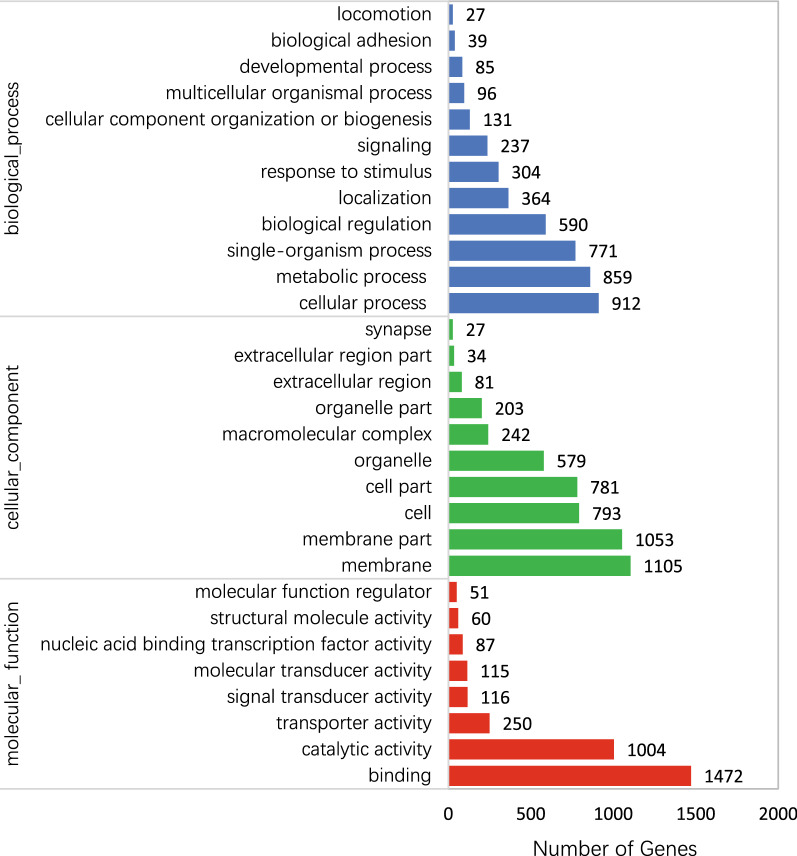
GO analysis on target genes of differently expressed miRNAs. Blue, green and red bars indicate biological processes, cellular components and molecular functions, respectively

In the GO function analysis processes, the biological functions related to the targets of the significantly differentially expressed miRNAs mainly included metabolic processes, cellular processes, membrane and membrane parts, catalytic activity and binding (Fig. 6). In addition, these targets may involve in the numerous KEGG pathway, such as facilitated glucose transporter, contactin-associated protein-like 2, lectin, mannose-binding 2, acetyl-CoA carboxylase/biotin carboxylase 1, bromodomain adjacent to zinc finger domain protein 2A and peroxidase (Additional file 4: Table S4).

## Discussion

A previous study demonstrated vertical transmission of ZIKV by *Ae. aegypti* mosquitoes from Jiegao and Mengding [25]. However, arbovirus infection of human beings mainly depends on horizontal transmission by mosquitoes. In the present study, we investigated the vector competence of these two *Ae. aegypti* mosquito strains to ZIKV with the aim to evaluate the risk of a ZIKA epidemic in the Jiegao and Mengding port cities of Yunan Province. We observed similar IR, DR and TR for the JG and MD strains under the same experiment condition, as well as the same propagation speed of the virus in these mosquitoes. ZIKV RNA was detected in the midgut and salivary gland of the JG and MD strains as early as 2 dpi, which is similar to results reported by Li et al. [30]; but the IR in these two tissues of the JG and MD strains were lower than those reported in the Ruili and Haikou strains by Li et al., possible due to the relative lower virus titer in the blood meal used in the latter study [30]. In our study, viral RNA was detected in the saliva of the two mosquito strains on 4 and 6 dpi, respectively. A previous study found that vector competence for DENV may be related to the genetic background of the mosquito, such as genes associated with the midgut escape barrier and, in particular, the quantitative trait loci (QTL) on chromosome III [31]. Therefore, we assume that the difference in arbovirus transmission competence between the two *Ae. aegypti* stains used in our study might be related to their different genetic backgrounds. CPE was observed on 7 dpi following inoculation of saliva samples collected from the JG and MD *Ae. aegypti* to C6/36 cells, which demonstrated infectious activity of ZIKV in the mosquito saliva. Due to the high vector competence of these two *Ae. aegypti* strains to ZIKV, our study of interactions between ZIKV and the mosquitoes will facilitate the development of prevention strategies to ZIKV. Therefore, we obtained small RNA profiles in the midgut and salivary gland of ZIKV-infected and non-infected *Ae. aegypti* mosquitoes through high-throughput sequencing and screened out the differently expressed miRNAs, which will provide valuable information for future studies on the molecular interactions between ZIKV and mosquitoes.

miRNAs play an essential role in host resistance to infections [32], and their expression levels in mosquitoes fluctuate in accordance with infections by *Plasmodium* parasites and viruses [33]. In our study, small RNA library analysis of ZIKV-infected *Ae. aegypti* midgut and salivary gland tissues identified 87 differentially regulated miRNAs for the JG strain and 216 differentially regulated miRNAs for the MD strain at different time points compared with the non-infected controls. Moreover, we observed the most significant differential expressions in miRNA profile in the 2-dpi libraries of both tissues from the two strains. A similar observation was reported by Saldaña et al*.* who used RNA extracted from whole mosquitoes for miRNA sequencing [18]. These authors reported that miRNA modulation in ZIKV-infected *Ae. aegypti* was also prominent in the early phase of 2 dpi, but they found fewer changes in the miRNA profile in the mosquitoes on day 14, with the highest viral load at this time point [18]. In another study, when *Ae. aegypti* was infected with the Ross River virus, a similar result was observed on 2 dpi [34]. These results highlight the potential importance of this period (2 dpi) during arbovirus infection and indicate that there is no positive correlation between changes in miRNA expression and viral load.

In the present study, further screening resulted in 27 and 74 miRNAs being selected from the miRNA expression profiles of ZIKV-infected and non-infected midgut and salivary gland tissues from JG and MD strain, respectively. Variability in transcriptomes was found between these different strains of *Ae. aegypti*, which indicates the need for caution in making generalizations about individual gene expression profiles across different strains of *Ae. aegypti* [35]. Thus, we hypothesize that the difference in the miRNA expression profile may be related to the different genetic backgrounds of the mosquitoes. The most significantly differentially expressed miRNAs of the midgut and salivary gland tissues from the two *Ae. aegypti* strains found in this study are aae-miR-2946 (log2 FC = 5.75, midgut, JG strain), aae-miR-989 (log2 FC = − 5.53, midgut, JG strain), aae-miR-286b (log2 FC = 4.32, salivary gland, JG strain), aae-miR-375 (log2 FC = − 4.79, salivary gland, JG strain); aae-miR-989 (log2 FC = 4.18, midgut, MD strain), aae-miR-957(log2 FC = 5.22, salivary gland, MD strain) and ae-miR-375 (log2 FC = − 4.62, salivary gland, MD strain). In other studies, those miRNAs were also reported after arboviruses infection. For example, aae-miR-375 was found to be mostly depleted after ZIKV infection [18], and it has also been shown to enhance DENV serotype 2 infection in an *Ae. aegypti* cell line [22]. In our study, aae-miR-375 showed the highest depletion in the salivary gland of two strains. Therefore, it will be interesting to experimentally test the function of this miRNA on ZIKV infection. Similarly, in the present study, the maximum FC was found in aae-miR-989 in the midgut. In comparison, Winter et al. reported that miR-989 also showed significant differential expression (log2 FC = 4) in *Plasmodium*-infected *Anopheles gambiae* and considered it to be a mosquito midgut-specific miRNA [36], whereas other studies found miR-989 to be rarely expressed in the midgut tissue of *Anopheles stephensi* [37] and exclusively expressed in the ovary of *An. gambiae* [38]. These contradictory conclusions may be due to the different mosquito species and viruses used in the different studies. In addition, aae-miR-989 is considered to be an important miRNA targeting mosquito immunity [39, 40]. Aae-miR-286b was downregulated in a study involving C6/36 *Ae. albopictus* cell line infected with DENV-2 [41]. In the present study, we report for the first time that aae-miR-2946 and aae-miR-957 showed significant differential expression after ZIKV blood meal in *Ae. aegypti*. To our knowledge, there have been no reports to date on the expression of these two miRNAs after ZIKV infection. We also found that aae-miR-2946, aae-miR-2941, aae-miR-989 and aae-miR-286b showed significant differential expression between both strains of the *Ae. aegypti* strains after the virus blood meal.

In terms of GO function analysis processes, the targets of the significantly differentially expressed miRNAs may be involved in metabolic processes, cellular processes, membrane and membrane parts and catalytic activity and binding, which indicates that the ZIKV–vector interaction is a complicated and highly regulated process. Recent research has shown that the metabolic conditions in the mosquito tissues play a critical role in the process of virus infection and replication and partially regulate virus transmission. Moreover, it has been demonstrated that ZIKV infection results in changes to the cellular metabolic environment, such as a significant enrichment of inosine and pseudo-uridine (Ψ) levels which may be related to RNA editing activity [42]. Therefore, future mechanistic studies can provide a new perspective to our understanding of the interactions between the ZIKV and its vector host.

## Conclusions

Both the JG and MD *Ae. aegypti* strains possess a strong vector competence to ZIKV. In this study, we compared miRNA profiles in the midgut and salivary gland of ZIKV-infected and non-infected *Ae. aegypti* mosquitoes. Differently expressed miRNAs were screened, which will provide valuable information for investigating the molecular mechanisms that influence the vector competence of mosquitoes. miRNAs regulate gene expression mainly by targeting cognate mRNAs for cleavage or translational repression. In a future study, we will seek to selected these targets of miRNAs by transcriptomics analysis or mRNA-Seq and validate the function in the regulation of mosquito competence, which will help to provide more efficient vector prevention strategies.

## Supplementary Information

**Additional file 1: Table S1.** Primers used for reverse transcription and quantitative PCR.

**Additional file 2: Table S2.** High-throughput sequencing profile of Small RNA.

**Additional file 3: Table S3.** The differentially expressed miRNAs based on small RNA-seq (|log2 FC|> 2).

**Additional file 4: Table S4.** Target genes predicted from differentially expressed miRNAs (|log2 FC|> 2).

## Data Availability

All data generated or analyzed during this study are included in this published article and its additional files.

## Abbreviations

cDNA: Complementary DNA
CHIKV: Chikungunya virus
CPE: Cytopathic effect
DENV: Dengue virus
DMEM: Dulbecco’s modified Eagle’s medium
dpi: Days post-infection
DR: Dissemination rate
FBS: Fetal bovine serum
FC: Fold change
FDR: False discovery rate
GO: Gene Ontology
IR: Infection rate
JG: Jiegao
KEGG: Kyoto Encyclopedia of Genes and Genomes
MD: Mengding
miRNA: MicroRNA
PAGE: Polyacrylamide gel electrophoresis
piRNA: P-element-induced wimpy testis-interacting RNA
QTL: Quantitative trait loci
RISC: RNA-induced silencing complex
RNAi: RNA interference
RNA-seq: RNA sequencing
ROS: Reactive oxygen species
rRNA: Ribosomal RNA
RT-PCR: reverse transcriptase-PCR
RT-qPCR: Reverse transcription-quantitative PCR
scRNA: Small cytoplasmic RNA
siRNA: Small interfering RNA
snoRNA: Small nucleolar RNA
snRNA: Small nuclear RNA
TE: Transmission efficiency
TR: Transmission rate
tRNA: Transfer RNA
ZIKV: Zika virus
