# Supplementary material for: Vector competence of Aedes aegypti and screening for differentially expressed microRNAs exposed to Zika virus
Source: Parasit Vectors. 2021 Sep 27;14:504. doi: 10.1186/s13071-021-05007-7 (PMC8477552; doi:10.1186/s13071-021-05007-7)
Supplement: Supplementary file 1 — Additional file 1: Table S1. Primers used for reverse transcription and quantitative PCR. [file 13071_2021_5007_MOESM1_ESM.doc]

**Table S1. Primers used for reverse transcription and quantitative PCR**

| Primers | miRNA | Sequence (5’-3’) |
| --- | --- | --- |
| RT primers | aae-miR-989 | GTCGTATCCAGTGCGTGTCGTGGAGTCGGCAATTGCACTGGATACGACGTACCA |
|  | aae-miR-2946 | GTCGTATCCAGTGCGTGTCGTGGAGTCGGCAATTGCACTGGATACGACACCCCA |
|  | aae-miR-2941 | GTCGTATCCAGTGCGTGTCGTGGAGTCGGCAATTGCACTGGATACGACCCGTGG |
|  | aae-miR-263a-5p | GTCGTATCCAGTGCGTGTCGTGGAGTCGGCAATTGCACTGGATACGACCCGTGAA |
|  | aae-miR-252-5p | GTCGTATCCAGTGCGTGTCGTGGAGTCGGCAATTGCACTGGATACGACCTCCTG |
|  | aae-miR-10 | GTCGTATCCAGTGCGTGTCGTGGAGTCGGCAATTGCACTGGATACGACAACAAATTC |
|  | aae-miR-375 | GTCGTATCCAGTGCGTGTCGTGGAGTCGGCAATTGCACTGGATACGACTAACTCG |
|  | aae-miR-RNU6B | GTCGTATCCAGTGCGTGTCGTGGAGTCGGCAATTGCACTGGATACGACAAAAATATGG |
| qPCR primers | aae-miR-989-F | GATgTgaTgTgacgTagTg |
|  | aae-miR-2946-F | GCTagTacggaaaagaTaTg |
|  | aae-miR-2941-F | TagTacggcTagaacTcca |
|  | aae-miR-263a-5p-F | GTaaTggcacTggaagaaTT |
|  | aae-miR-252-5p-F | TTaagTacTagTgccgcag |
|  | aae-miR-10-F | GCacccTgTagaTccgaa |
|  | aae-miR-375-F | GATTTgTTcgTTTggcTcg |
|  | universal reverse primer | TGCGTGTCGTGGAGTC |
