# Supplementary material for: Vector competence of Aedes aegypti and screening for differentially expressed microRNAs exposed to Zika virus
Source: Parasit Vectors. 2021 Sep 27;14:504. doi: 10.1186/s13071-021-05007-7 (PMC8477552; doi:10.1186/s13071-021-05007-7)
Supplement: Supplementary file 2 — Additional file 2: Table S2. High-throughput sequencing profile of Small RNA. [file 13071_2021_5007_MOESM2_ESM.docx]

**Table S2. High throughput sequencing profile of Small RNA**

| Sample name | Raw tag count | Clean tag count | Percentage of clean tag (%) | Mapped tag | Percentage  (%) |
| --- | --- | --- | --- | --- | --- |
| J_2d_m_c | 30379746 | 23869311 | 78.57 | 20753348 | 86.95 |
| J_2d_m_zikv | 32876712 | 23868984 | 72.6 | 20351408 | 85.26 |
| J_2d_sg_c | 27862809 | 24654652 | 88.49 | 21486563 | 87.15 |
| J_2d_sg_zikv | 28571428 | 23883013 | 83.59 | 19733028 | 82.62 |
| J_4d_m_c | 30379746 | 24003916 | 79.01 | 20541157 | 85.57 |
| J_4d_m_zikv | 29268292 | 23945749 | 81.81 | 20189732 | 84.31 |
| J_4d_sg_c | 29629629 | 24085743 | 81.29 | 19545441 | 81.15 |
| J_4d_sg_zikv | 30769230 | 24046239 | 78.15 | 19459070 | 80.92 |
| J_6d_m_c | 29822378 | 26144193 | 87.67 | 22172417 | 84.81 |
| J_6d_m_zikv | 28304321 | 24060233 | 85.01 | 20919201 | 86.95 |
| J_6d_sg_c | 29481027 | 25159165 | 85.34 | 19639733 | 78.06 |
| J_6d_sg_zikv | 31168831 | 24140854 | 77.45 | 19494911 | 80.75 |
| M_2d_m_c | 35820895 | 23935059 | 66.82 | 15748048 | 65.79 |
| M_2d_m_zikv | 28335688 | 24676729 | 87.09 | 17752186 | 71.94 |
| M_2d_sg_c | 27917462 | 24927969 | 89.29 | 16543592 | 66.37 |
| M_2d_sg_zikv | 28884627 | 25485225 | 88.23 | 20867641 | 81.88 |
| M_4d_m_c | 28915662 | 24056395 | 83.2 | 21679245 | 90.12 |
| M_4d_m_zikv | 28815760 | 24195939 | 83.97 | 22017579 | 91 |
| M_4d_sg_c | 29268292 | 24093410 | 82.32 | 20227742 | 83.96 |
| M_4d_sg_zikv | 29382706 | 24186834 | 82.32 | 19810516 | 81.91 |
| M_6d_m_c | 30769230 | 24064704 | 78.21 | 20272628 | 84.24 |
| M_6d_m_zikv | 28651198 | 26012839 | 90.79 | 23227413 | 89.29 |
| M_6d_sg_c | 29268292 | 24014185 | 82.05 | 18865484 | 78.56 |
| M_6d_sg_zikv | 32000000 | 24107143 | 75.33 | 18825231 | 78.09 |
